# Private prescribing of controlled opioids in England, 2014–2021: a retrospective observational study

**DOI:** 10.3399/BJGP.2023.0146

**Published:** 2023-11-14

**Authors:** Isabella Martus, Brian MacKenna, William Rial, Jon Hayhurst, Georgia C Richards

**Affiliations:** Oxford Medical School, Medical Sciences Divisional Office, University of Oxford, Oxford.; Bennett Institute for Applied Data Science, Nuffield Department of Primary Care Health Sciences, University of Oxford, Oxford; specialist pharmacist, NHS England.; Department of Clinical, Pharmaceutical and Biological Science, University of Hertfordshire, Hatfield; regional chief pharmacist, East of England, NHS England.; NHS England, South West Region.; Centre for Evidence-Based Medicine, Nuffield Department of Primary Care Health Sciences, University of Oxford, Oxford.

**Keywords:** controlled drugs, dependence, opioids, patient safety, primary health care, private prescribing

## Abstract

**Background:**

Trends in NHS opioid prescribing have been well published, yet trends in private prescribing of opioids have not been widely established.

**Aim:**

To assess trends and geographical variation in controlled opioids prescribed by private prescribers in England.

**Design and setting:**

This was a retrospective observational study in English primary health care.

**Method:**

Data on Schedule 2 and 3 controlled opioids (‘controlled opioids’) were obtained from the NHS Business Services Authority (BSA) using Freedom of Information (FOI) requests between 1 January 2014 and 30 November 2021. Absolute counts and rates of the number of items dispensed per cumulative number of registered private prescribers were calculated and stratified over time, by opioid type, and geographical region.

**Results:**

This study found that 128 341 items of controlled opioids were prescribed by private prescribers in England between January 2014 and November 2021, which decreased by 50% from 23 339 items (4.09 items/prescriber) in 2014 to 11 573 items (1.49 items/prescriber) in 2020. Methadone (36%, *n* = 46 660) was the most common controlled opioid prescribed privately, followed by morphine (18%, *n* = 22 543), buprenorphine (16%, *n* = 20 521), and oxycodone (12%, *n* = 15 319). Prescriptions were highest in London (74%, *n* = 94 438), followed by the South-East of England (7%, *n* = 9237). A proportion of items (*n* = 462; 0.36%) were prescribed by ‘unidentified doctors’ where the prescription is not readily attributable to an individual prescriber by the BSA.

**Conclusion:**

Controlled opioids prescribed by private prescribers in England decreased and were primarily prescribed in London. To ensure patient safety, the monitoring and surveillance of controlled opioids dispensed privately should continue and items linked to ‘unidentified doctors’ should be addressed further.

## INTRODUCTION

Opioids are strong analgesics often prescribed in primary care for chronic pain.^1^^–^3 There is strong evidence to suggest that the harms of opioids outweigh the benefits when used at high doses and for long durations.^4^^,^^5^ Over the past three decades, opioid prescribing has increased in the UK,^1^^,^^6^ as well as the subsequent increase in opioid dependence, overdose, and deaths associated with their use.^7^^,^^8^

Studies on the use of opioids in England have focused on prescriptions dispensed in the NHS or qualitative studies of prescribers in the community,^1^^–^3 despite opioids also being available to purchase over-the-counter (for example, codeine linctus and co-codamol), from online pharmacies or the ‘dark web’, and through prescriptions from private prescribers.^9^^–^11 In an analysis of over-the-counter codeine sales in 31 countries, the UK was found to purchase the fourth most products containing codeine.^9^ Preventable deaths from purchasing medicines online, including opioids, have been reported in England and Wales, and an analysis of online marketplaces for controlled substances has found a wide variety and availability of opioids in the UK.^10^^,^^12^ The Care Quality Commission’s (CQC) report on the safer management of controlled drugs briefly mentions trends of some controlled opioids from both NHS and private prescribers, but provides no long-term assessment or insights into geographical variation.^13^^–^15 A national survey of community pharmacies in 1995 assessed primary and secondary NHS and private methadone prescriptions.^11^ The findings from that survey described key differences between NHS and private prescribing of opioids, including an increased dispensing of methadone in tablet form (33% private versus 10.9% NHS) and larger-quantity provisions privately rather than daily dispensing in the NHS. The insights from this research would not have been possible if using NHS prescribing alone. Therefore, an up-to-date analysis of opioids dispensed privately is required to better understand the use of opioids in England.

In England, the majority of the population access health care and services publicly through the NHS, with an estimated 570 million patient contacts in 2021/2022 alone.^16^ Private health care is provided by a hospital, clinic, or provider that is independent but complementary to the NHS.^17^ It is paid for by the individual, either directly or through privately funded medical insurance. Waiting times and timely access to NHS services is an issue, which has increased the number of people turning to private services for timely access to care.^18^ However, there remains a risk that patients may obtain prescriptions concurrently from NHS and private prescribers, or multiple private prescribers.

**Table:** How this fits in

There are concerns over the long-term, high-dose use of opioids in people with chronic pain — trends for which had not yet been described outside of the NHS. This retrospective observational study aimed to explore the volume of opioids supplied in the private community setting in England, using Freedom of Information (FOI) requests sent to the NHS Business Services Authority (BSA). The volume of controlled opioid items prescribed by private prescribers halved in England between January 2014 and November 2021, with three-quarters prescribed in London. Although Controlled Drugs Accountable Officers (CDACs) are responsible for managing controlled opioids, increasing access to non-NHS data, including data on the prescribing of controlled opioids in the private sector, will improve the safety, monitoring, and surveillance of opioids, which will help identify harms and improve patient care.

Opioids used in health care are controlled under the Misuse of Drugs Act 1971 and the Misuse of Drugs Regulations 2001 as they have the potential to cause harm and are thus subject to increased controls. The legislation aims to prevent the misuse of controlled drugs and the Regulations allocate controlled drugs, including gabapentinoids and opioids, into schedules (1 to 5) that set out the controls associated with each schedule.^19^ These include the specific requirements for private prescriptions for Schedule 2 and 3 controlled drugs. For Schedule 2 and 3 controlled drugs the Shipman Inquiry made several recommendations on the prescribing and monitoring of controlled drugs,^20^ with one including the introduction of private controlled drug prescriber practitioner codes.^21^ The Controlled Drugs (Supervision of Management and Use) Regulations 2013 sets out the monitoring requirements. Since 2007, private prescribers in England must write prescriptions for Schedule 2 and 3 controlled drugs on a special prescription form, allowing data to be captured (Box 1).^22^ Yet, an analysis has not been openly published, to the authors’ knowledge, to share such data on the recent trends of controlled opioids dispensed by private prescribers. Therefore, the aim of this study was to evaluate trends and geographical variation of controlled opioids prescribed by private prescribers in the English community.

**Box 1.** A summary of how private prescription data for Schedule 2 and 3 controlled drugs are collected from private prescribers by the NHS Business Services Authority (BSA)

Misuse of Drugs Regulations 2001 mandates private prescriptions of Schedule 2 and 3 controlled drugs are written on controlled stationery forms that carry a unique prescriber identification number (PIN). Prescribers wishing to prescribe schedule 2 and/or schedule 3 controlled drugs in private practice are assigned a PIN by the NHS BSA following an application process, currently managed by NHS England controlled drug accountable officers (CDAO). NHS prescribers have an NHS prescriber number but can also apply for a PIN. NHS Digital publishes the data on PINs, which are available for supplying pharmacies to search. The local lead CDAO can also be contacted to confirm whether a prescriber has a PIN and if it is current. The requisite prescription forms (FP10PCD) are obtained by the prescriber through Primary Care Support England. Prescription for controlled drugs is written by private prescriber with PIN applied. The pharmacy dispenses medication, and the patient signs the back of the prescription form at the pharmacy. Forms with unique PINs are submitted to NHS BSA from the pharmacy, monthly in arrears. Forms scanned by NHS BSA capture information including prescriber PIN and item prescribed. Prescription forms sent to NHS prescription services and included in various internal reports by NHS BSA are included in EPACT2 but with restricted access.^28^

## METHOD

### Study design

The authors designed a retrospective observational study and preregistered the study protocol on an open repository.^23^

### Data sources

The authors obtained data from the NHS Business Services Authority (BSA) using three Freedom of Information (FOI) requests to acquire the most up-to-date data (ePACT2, NHS BSA, Copyright 2022; this information is licensed under the terms of the Open Government License).^24^^–^26 ePact is Oracle visualisation software that BSA uses to allow users to access prescription data from NHS BSA, which is sourced from the NHS BSA Data Warehouse and is derived from products prescribed and dispensed in the community in England.^24^^–^26 Data for Wales, Scotland, and Northern Ireland are collected and stored locally. The data summarises the number of controlled opioid prescriptions (‘items’), which are Schedule 2 and 3 drugs, dispensed for England at the area team levels (2014–2019) and the sustainability and transformation partnerships (STP) (2020–2021) regions for the British National Formulary (BNF) Chemical Substances under section 7.4.2, in quarterly splits between January 2014 and November 2021.

Data on the number of registered private prescribers in England were obtained from NHS Digital, which had a record of private controlled drug prescribers in England since 2006.^27^

### Data analysis

The authors combined the three FOI requests and data on the number of registered private prescribers in England in a Google Sheet that was used for analysis. First, the total crude number of controlled opioid items dispensed between 1 January 2014 and 30 November 2021 was calculated and this count was stratified by year. For this calculation, a total range is presented as for the second FOI request for 2019 data; NHS BSA did not provide values if the number of items dispensed were below five.^25^ Thus a total range was estimated for the current study using the lowest estimate of one item and the highest estimate of four items. In all figures and other calculations, the lowest estimate has been used. The absolute percentage change over time was calculated between January 2014 and December 2020 because the authors received 11 months of data for 2021.

To control for changes in the population over time, the number of registered private prescribers was used as a proxy to calculate the rate. To calculate the rate, the authors summed the number of registered private prescribers in England from 2006 until 2014 and then cumulatively until 2021. The authors divided the absolute number of items by this figure to determine the rate of controlled opioid items dispensed per registered private prescriber in England. For this calculation, it was assumed that all registered private prescribers were active.

To assess types of opioids, the authors combined similar BNF Chemical Substances regardless of the salt formulation (for example, data on ‘morphine sulfate’ were combined with ‘morphine’) or non-opioid medicine in the formulation (for example, data on ‘buprenorphine hydrochloride/naloxone hydrochloride’ were combined with ‘buprenorphine’). The authors summed the types of opioids and calculated a percentage of the total for each type and the percentage change over time.

To examine geographical variation, the authors allocated each area team level (2014–2019) and STPs (2020–2021) to one of the nine regions of England, including London, East of England, East Midlands, West Midlands, North East of England, North West of England, Yorkshire and Humber, South East of England, and South West of England. The number of items of controlled opioids dispensed in each region was summed and deciles were used to generate a choropleth map. For the latest data (1 January to 30 November 2021), deciles and a choropleth map were used to illustrate the number of items dispensed across STP regions. Percentages were calculated to determine the areas with the greatest and lowest dispensing.

### Software and data sharing

Google Sheets was used to process and analyse the data and to produce line and bar graphs. DataWrapper was used to produce the choropleth map (https://www.datawrapper.de). The study protocol, data, and materials are openly available via the Open Science Framework.^23^^,^^29^

## RESULTS

**Figure 1.**
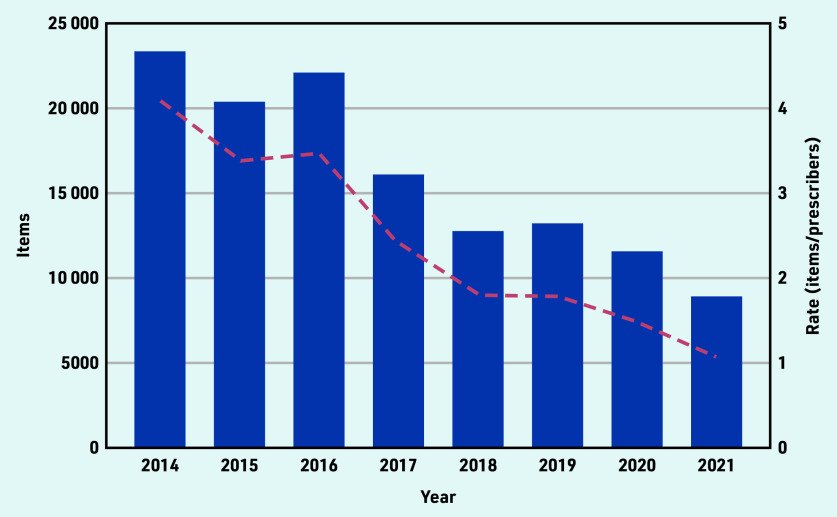
*Number of controlled opioid items (left* y*-axis) dispensed by private prescribers in England over time from 1 January 2014 to 30 November 2021 and the rate of items dispensed by the cumulative number of registered private prescribers (red dashed trend line).*

Between 128 341 and 129 040 controlled opioid items were dispensed by private prescribers in England between January 2014 and November 2021. The volume of controlled opioids prescribed by private prescribers decreased by 50% between January 2014 (*n* = 23 339 items) and December 2020 (*n* = 11 573 items) (Figure 1). Controlling for the number of private prescribers in England, trends decreased by 64% from 4.09 items/private prescriber in 2014 to 1.49 items/prescriber in 2020 (Figure 1; Supplementary Table S1).

**Figure 2.**
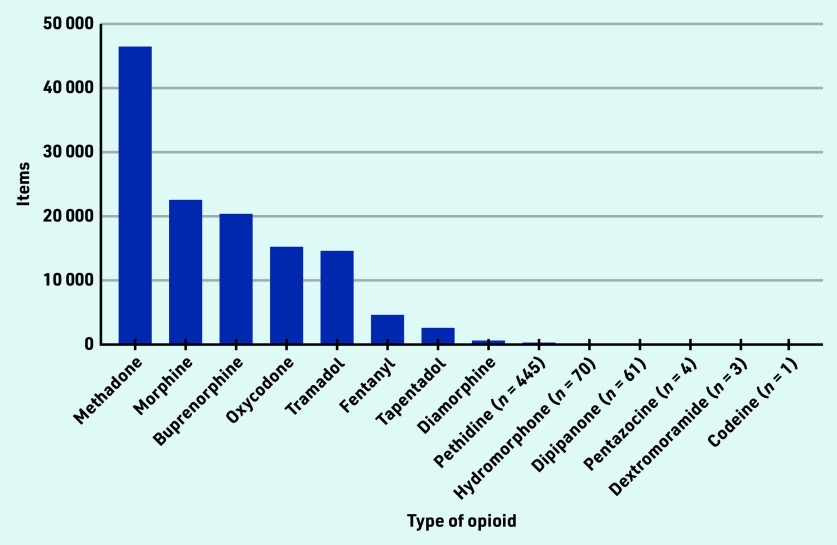
*Types of controlled opioids dispensed by registered private prescribers in England between January 2014 and November 2021.*

There were 14 different types of opioids that were dispensed by private prescribers in England (Figure 2). Only codeine preparations for injection are controlled opioids (Schedule 2). Codeine was included by NHS BSA in the first FOI request (2014– 2018), but only appeared once in the second quarter of 2018.^29^ Methadone was the most common controlled opioid dispensed (36%; *n* = 46 660 items), followed by morphine (18%; *n* = 22 543 items), buprenorphine (16%, *n* = 20 521 items), oxycodone (12%; *n* = 15 319 items), and tramadol (11%; *n* = 14 686 items) (Figure 2).

**Figure 3.**
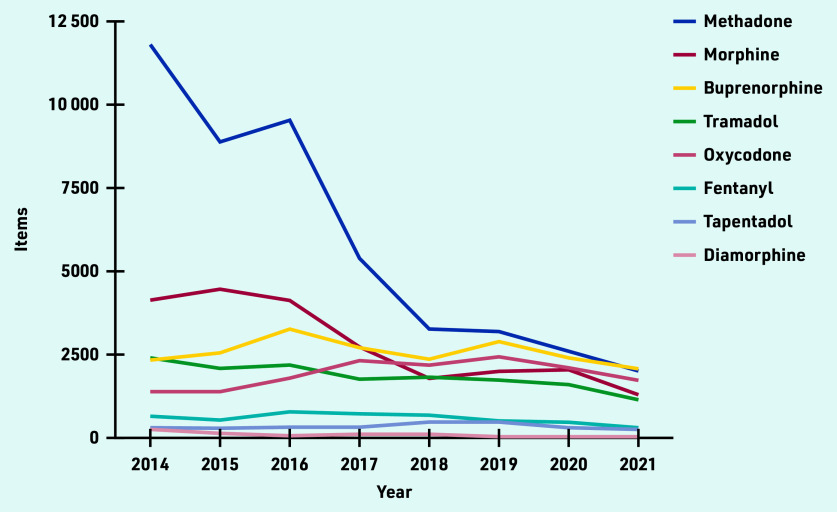
*Trends of the top eight most common opioids dispensed by registered private prescribers in England from January 2014 to November 2021.*

Over time, the number of items dispensed decreased for most types of opioids, except for oxycodone (Figure 3) and hydromorphone (Supplementary Table S2), which both increased. Five types of opioids, methadone, morphine, buprenorphine, oxycodone, and tramadol, represented 93% (*n* = 119 729 items) of all controlled opioids dispensed in England between 2014 and November 2021.

**Figure 4.**
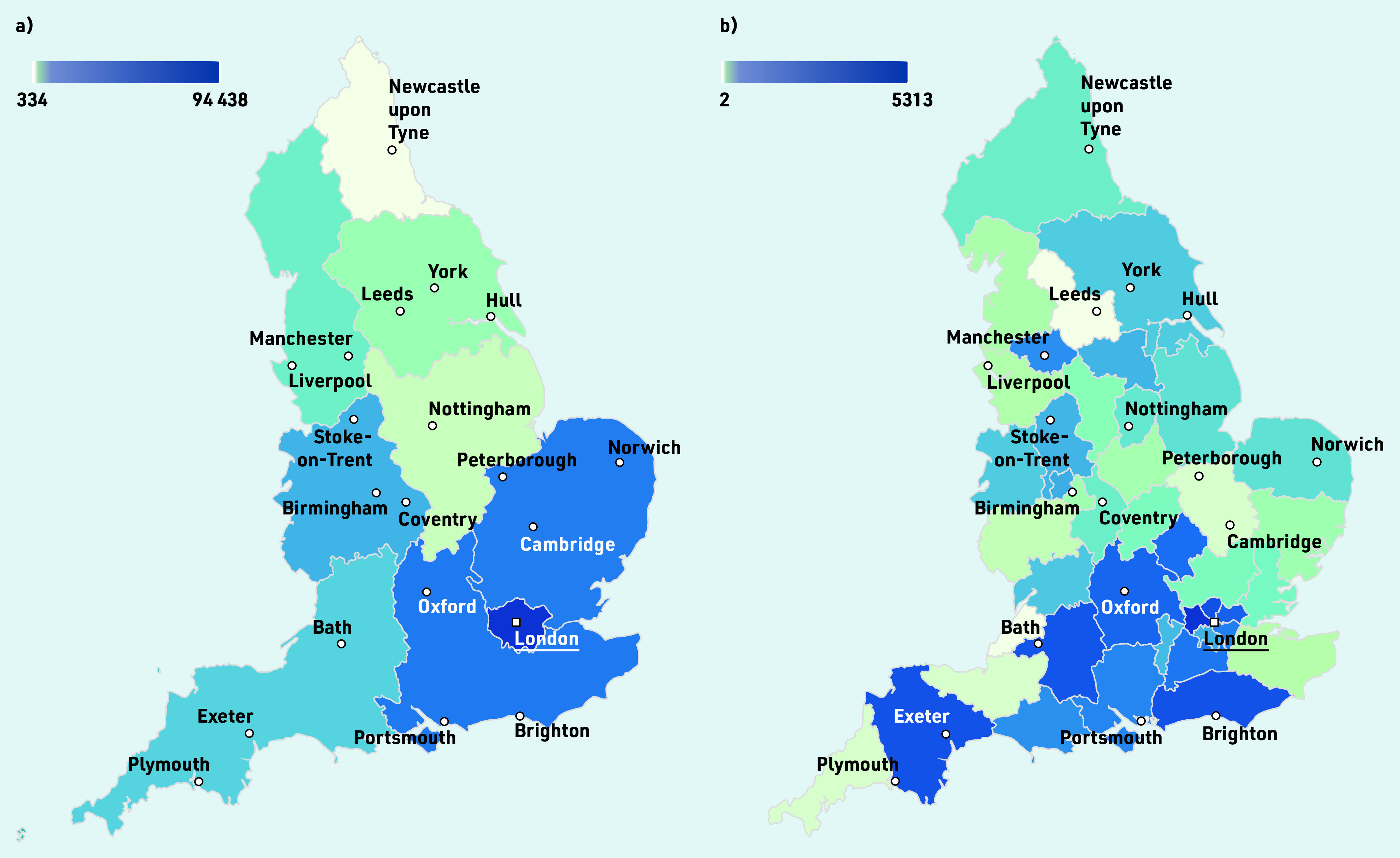
*Geographical variation of the absolute number of controlled opioid items dispensed by private prescribers in England, created using deciles in Datawrapper. a) Represents the total number of items across the nine regions of England between 1 January 2014 and 30 November 2021. b) Shows the number of items dispensed in each of the sustainability and transformation plans (STP) region for the latest data period of 1 January to 30 November 2021. The number of items dispensed from unidentified doctors is not included in these maps.*

Most controlled opioid items were dispensed in London (74.6%; *n* = 94 438 items) followed by the Southeast (7.2%, *n* = 9237) and Eastern England (6.7%, *n* = 8644) (Figure 4a). There were only a few items dispensed in the Northeast of England (0.26%; *n* = 334) and East Midlands (0.69%; *n* = 886) (Supplementary Table S3).

For the latest data (1 January to 30 November 2021), the North West of London (*n* = 5313) and Staffordshire and Stoke on Trent (*n* = 571) dispensed the most items of opioids, whereas West Yorkshire and Harrogate Health and Care Partnership (*n* = 2) and Somerset (*n* = 2) were in the lowest deciles (Figure 4b; Supplementary Table S4).

There were 462 items (0.36%) of controlled opioids that were attributed to ‘unidentified prescribers’ by NHS BSA in 2015 and between January 2018 and November 2021, described as ‘items that could not be allocated to an individual prescriber’.^24^^,^^26^^,^^29^ Over 53% of these (246 items) were allocated to unidentified doctors in 2021 alone. An ‘unidentified prescriber’ can occur when the details printed or written on a prescription do not exactly match those held by the NHS BSA, but NHS BSA can visually inspect the form to determine the prescriber. Morphine (25%; *n* = 117 items), buprenorphine (24%; *n* = 109 items), and methadone (17%; *n* = 79 items) were the most common types of opioids that were attributed to ‘unidentified prescribers’ (Supplementary Table S5).

## DISCUSSION

Controlled opioids prescribed by private prescribers in England decreased between January 2014 and November 2021. Three- quarters of privately prescribed controlled opioids were from prescribers in London, with methadone and morphine being the most common types of opioids. A small proportion of controlled opioids dispensed privately were attributed to ‘unidentified prescribers’ by NHS BSA.

### Strengths and limitations

A previous study that examined the use of privately prescribed opioids in England was conducted in 1995,^11^ therefore the current study provides a much needed update on the trends and geographical variations of controlled opioids dispensed by private prescribers in England. In comparison with previous research that used a sample of data from community pharmacies over 1 year and focused on injectable methadone for people with opioid addiction,^11^ the current study examined all types of controlled opioids over 8 years.

Electronic prescribing of Schedule 2 and 3 controlled drugs by private prescription is not permitted so the data capture remains a manual process. Without access to electronic health records, it is also not possible to assess the doses, duration, or indication of use. Instead, the data represented the number of times that a controlled opioid appeared on the prescription form. In the FOI that provided data for 2019, the number of items dispensed were retracted by NHS BSA if the total was less than five. The authors of the current study therefore estimated the total items using an upper and lower (four or one) figure for 2019.

As it is not known what proportion of the English population visited private prescribers over the study period, the trends were standardised by the cumulative number of registered private prescribers in England. This is a limitation that restricts comparison of private prescribing with the NHS prescribing of opioids as the two datasets have a different denominator. Importantly, the data in the current study only reflect opioids deemed controlled substances in Schedule 2 and 3 during the study period (January 2014 and November 2021). Thus the findings do not represent all opioids obtained from private prescribers in England. For example, tramadol was reclassified as a Schedule 3 controlled drug in April 2014, thus the authors could only receive data for tramadol from this point forward. Opioids in Schedule 4 and 5, which are not subject to the same prescription and monitoring requirements, such as some codeine preparations and oral morphine solution 10 mg/5 mL (Oramorph), were not able to be captured in this study. However, the NHS BSA included one item of codeine in the 2014–2018 dataset.^25^

### Comparison with existing literature

There has been limited information available on the trends of private drug prescribing in England. Opioid research has therefore focused on NHS prescribing,^1^^,^^6^^,^^30^ which, similarly to the current study, found that the prescribing of opioids started to decrease in 2016.^1^ There is ongoing research that is investigating more recent trends in opioid prescribing between January 2018 to March 2022 using OpenSafely.^31^ A report on the long-term (>3 months) use of opioids in England found that prescribing rose during the COVID-19 pandemic.^32^ However, the overall trend of the number of items dispensed in the community decreased between 2016 and 2021 in England.^33^

The CQC’s annual reports on the safer management of controlled drugs similarly found that prescription methadone, fentanyl, morphine, and tramadol decreased between 2020 and 2021,^14^^,^^15^ and that methadone was the most commonly privately prescribed controlled drug in 2014.^34^ In the current study, oxycodone and hydromorphone were the only types of opioid that increased in volume during the study period. A retrospective study that assessed the prescribing of oxycodone in the English NHS between 2013 and 2018 similarly found an increase in the median rate of oxycodone prescriptions per 1000 population.^35^ Methadone, which was the most common opioid prescribed by private prescribers in the current study, is often used for opioid substitution therapy. Thus, this high use in private practice may be driven by the lack of access to appropriate services and significant regional variation in drug misuse services in England.^36^

Previous studies have found geographical variation in the NHS prescribing of opioids across England. In the North of England and in areas of social deprivation, higher rates of opioids have been prescribed.^1^^,^^8^ In contrast, the current study found that controlled opioids were prescribed more often from London compared with the North of England. The use of private services located in London may be driven by the availability of private clinics and hospitals, the population density, the needs of commuters and visitors to London that require convenient and quick access to health care,^37^ as well as the higher wages that may contribute to the affordability of private doctors in London.^38^ However, as it is not possible to determine the number of people receiving controlled opioids prescriptions from private prescribers, it is also not possible to establish where they are residents.

### Implications for research and practice

Information on private prescribing is not routinely collected or published as it does not relate to publicly funded health care. However, this study has illustrated that examining data on controlled opioids dispensed in the private setting can provide a more comprehensive overview of the total volume of opioids being used in England. The finding that prescribing of controlled opioids decreased is in line with the UK’s National Institute for Health and Care Excellence guidelines for chronic primary pain in >16-year-olds, which advises against the use of opioids.^39^ However, the 462 items linked to ‘unidentified doctors’ has potential implications for patient safety that should be resolved. Furthermore, codeine, which may not be a Schedule 2 or 3 controlled drug depending on the preparation, was included in one of the datasets that should be examined.

Although the authors were able to access the data through FOI requests, NHS BSA should consider making such data on all controlled drugs publicly available without the need for an FOI. If openly available, these data could be integrated into the NHS BSA’s opioid comparators,^40^ and other services such as openprescribing.net who utilise NHS BSA data.^41^ This would improve the routine surveillance of opioids, support the analysis of private controlled opioid prescribing over time, and allow for the impact assessment of guidelines and regulations relating to controlled opioids. In the data provided by NHS BSA, the total number of items were redacted if below five for 2019 only. As this was not the case for all other years, NHS BSA should standardise their approach so data are consistent across all requests.

In conclusion, the private prescribing of controlled opioids in England has decreased. There was geographical variation with the majority of controlled opioids prescribed privately from London. The data used in the current study were obtained using FOI requests, which should be made available and accessible to improve the surveillance of controlled opioids. The current findings provide an important insight into another avenue by which people obtain controlled opioids in the community.

## Data

All study materials and data are openly available via online repositories (https://doi.org/10.17605/OSF.IO/HFKDQ). The study protocol was preregistered on the Open Science Framework (https://doi.org/10.17605/OSF.IO/HCKPU).^23^ Data were obtained from the NHS Business Services Authority (BSA) through Freedom of Information requests; ‘ePACT2, NHS BSA Copyright 2022’. This information is licensed under the terms of the Open Government Licence.
